# Distributional genetic effects reveal context-dependent molecular regulation in human brain aging and Alzheimer’s disease

**DOI:** 10.21203/rs.3.rs-8219833/v1

**Published:** 2025-12-01

**Authors:** Anjing Liu, Roulan Jiang, Ruixi Li, Xuewei Cao, Zining Qi, Ru Feng, Hao Sun, Masashi Fujita, Natacha Comandante-Lou, Chirag M Lakhani, Jenny Empawi, David A. Knowles, Xiaoling Zhang, Kushal K. Dey, Philip De Jager, David Bennett, The Alzheimer’s Disease Functional Genomics Consortium, Tianying Wang, Gao Wang

**Affiliations:** 1 Center for Statistical Genetics, The Gertrude H. Sergievsky Center, Columbia University, New York, NY, USA.; 2 Department of Statistics and Data Science, Tsinghua University, Beijing, China; 3 Computational and System Biology, Sloan Kettering Institute, Memorial Sloan Kettering Cancer Center, New York, NY, USA.; 4 Division of Genetic Medicine, Department of Medicine, The University of Chicago, Chicago, IL, USA; 5 Graduate School of Biomedical Sciences, Icahn School of Medicine at Mount Sinai, New York, NY, USA; 6 Center for Translational & Computational Neuroimmunology, Columbia University, New York, NY, USA; 7 New York Genome Center, New York, NY, USA.; 8 Department of Biomedical Genetics, Boston University Chobanian & Avedisian School of Medicine, Boston, MA, USA.; 9 Department of Computer Science, Columbia University, New York, NY, USA.; 10 Department of Systems Biology, Columbia University, New York, NY, USA.; 11 Data Science Institute, Columbia University, New York, NY, USA.; 12 Department of Biostatistics, Boston University School of Public Health, Boston, MA, USA; 13 Physiology, Biophysics and System Biology, Weill Cornell Medicine, New York, NY, USA.; 14 Gerstner Sloan Kettering Graduate School of Biomedical Sciences, New York, NY, USA.; 15 Taub Institute for Research on Alzheimer’s Disease and the Aging Brain, Columbia University, New York, NY, USA; 16 Department of Neurology, Columbia University, New York, NY, USA.; 17 Rush Alzheimer’s Disease Center and Department of Neurological Sciences, Rush University Medical Center, Chicago, IL, USA; 18 Department of Statistics, Colorado State University, Fort Collins, CO, USA; 19 Department of Biostatistics, Columbia University, New York, NY, USA

## Abstract

Molecular QTL studies quantify whether genetic variants affect molecular traits, but non-linear effects including distributional patterns, variance, and interactions provide mechanistic insights beyond mean-level associations. Methods for detecting distributional effects have been developed for eQTL analysis, yet applications have focused on method demonstrations rather than large-scale biological discovery. We comprehensively mapped quantile, variance, and interaction QTLs across 34 data-set from 22 molecular contexts in >2,300 human brain donors, revealing that 48.7% of quantile QTLs (qQTLs) exhibit context-dependent regulation invisible to linear models, with enrichment at phenotypic extremes and in cell-type-specific regulatory elements, chromatin accessibility regions, and long-range chromosomal contacts. qQTL variants explained additional trait heritability beyond linear QTLs for brain-related traits. At Alzheimer’s disease (AD) risk loci, qQTL analysis revealed complex regulatory architecture including variance effects at *PITRM1*, lower-quantile-specific effects at *TMEM106B* partially explained by *APOE* ε4 interactions, and coordinated epigenetic regulation at loci harboring *CHRNE*/*SCIMP*/*RABEP1*. Quantile-based transcriptome-wide association studies identified 34 AD risk genes and additional aging-related genes beyond standard TWAS, with enrichment in immune regulation and telomere maintenance pathways where distributional effects may reflect threshold-dependent mechanisms. Our non-linear QTL atlas and qTWAS resource enable characterization of context-dependent regulatory effects in complex disease genetics.

## INTRODUCTION

Traditional molecular QTL (xQTL) mapping asks whether genetic variants affect gene or protein expression, treating this largely as a detection question rather than characterizing effect size patterns that reveal regulatory mechanisms. Linear regression identifies variants associated with mean expression changes, yet a single effect size estimate captures limited mechanistic information: variants may operate primarily at phenotypic extremes, exert effects that vary across cellular contexts, or alter expression variability rather than central tendency. While conventional eQTL and pQTL analyses have successfully linked genetic variants to expression changes and explained portions of GWAS signals, effect size patterns across expression distributions remain largely uncharacterized even in large-scale efforts like the Genotype-Tissue Expression (GTEx) project^1^. As genome-wide association studies continue to identify hundreds of loci for complex diseases, particularly for the >95% of variants in non-coding regions, the gap between genetic association and mechanistic understanding underscores the need for analytical frameworks that characterize the full spectrum of regulatory variation beyond mean effects^2,3^.

Mapping non-linear genetic effects offers a potential solution to characterize this regulatory complexity. Current approaches include interaction QTLs (iQTLs), which test for context-dependent regulation such as sex-specific, age-dependent, or cell-type-specific effects and can estimate context-specific effect sizes, but require *a priori* specification of interacting factors and employ parametric interaction models (e.g., G + E + G×E terms)^4^. Variance QTLs (vQTLs) identify genetic variants associated with expression variability and can capture latent interactions without predefined factors, but detect variance effects without estimating how effect sizes vary across phenotypic distributions^5,6^. Additionally, both approaches have shown limited statistical power in existing molecular QTL studies, restricting discovery of non-linear regulatory effects, particularly in disease-relevant tissues such as the human brain where emerging cellular resolution datasets enable investigation of regulatory heterogeneity that may reflect non-linear genetic mechanisms^7,8^.

Quantile regression offers a complementary framework by modeling how variants influence the entire distribution of molecular phenotypes rather than just the mean, enabling both detection of non-linear effects and characterization of effect size patterns across phenotypic distributions^9^. Unlike linear models, quantile QTL (qQTL) analysis can identify variants with heterogeneous effects across the phenotype distribution and quantify how effect sizes vary at different expression levels, capturing regulatory mechanisms that operate primarily at expression extremes or that vary across unobserved biological contexts. Methods for detecting distributional effects have been developed, yet applications focus on method demonstrations rather than large-scale biological discovery^10,11^. The potential for qQTL to reveal regulatory architecture missed by standard approaches, particularly in the context of aging and neurodegeneration, and its systematic integration with disease-specific GWAS for gene prioritization remain largely unexplored.

Here, we present an atlas of non-linear molecular QTLs in the aging human brain, generated from >2,300 brain donors across 28 molecular contexts spanning bulk and single-nucleus gene expression and protein abundance. We demonstrate that qQTL analysis identifies context-dependent regulatory effects complementary to those captured by linear models, with approximately 48.7% of brain qQTLs showing either no significant linear association or strong quantile specific effects not captured by xQTL. Functionally, qQTL variants show enhanced enrichment in distal cell-type-specific regulatory elements and explain additional GWAS heritability for brain-related traits beyond linear QTLs. We dissect distributional regulatory effects at established Alzheimer’s Disease (AD) risk genes including *PITRM1*, *TMEM106B*, and the *CHRNE*/*SCIMP*/*RABEP1* cluster, revealing complex context-dependent regulatory architecture missed by linear analysis. Through systematic comparison with iQTL and vQTL approaches, we show that qQTL captures variance effects as a special case and provides complementary power for detecting interaction effects, particularly in *counteracting interactions* where linear regression shows limited sensitivity. Finally, we introduce quantile-based transcriptome-wide association studies (qTWAS) and demonstrate its utility for gene prioritization in AD and aging, identifying 34 novel AD gene associations and additional aging-related genes not detected by standard TWAS approaches. Our study establishes qQTL as a powerful and complementary approach to linear QTL analysis, and we provide brain qTWAS weights as a resource for broad application across complex disease studies.

## RESULTS

### Multi-omics non-linear QTL discovery and disease integration in human brain

To systematically characterize non-linear genetic regulatory effects in the human brain, we applied quantile regression to 22 datasets from the FunGen-xQTL project of the Alzheimer’s Disease Functional Genomics Consortium, spanning bulk tissue expression QTLs from 9 brain regions, single-nucleus expression QTLs from 6 cell types in DLPFC, and protein QTLs from 3 brain regions, derived from four cohorts: Religious Orders Study/Memory and Aging Project (ROSMAP, n=777 bulk tissue RNA expression, n=737 single-nucleus, n=540 proteome, n=230 monocytes from plasma), Mount Sinai Brain Bank (MSBB, n=274 bulk tissue RNA expression, n=184 proteome), Charles F. and Joanne Knight Alzheimer’s Disease Research Center (Knight-ADRC, n=354 bulk tissue RNA expression, n=412 proteome), and Microglia Genome Atlas (MiGA, n=66 microglia)^12–17^ (**Table S1**). All molecular phenotypes and genotypes underwent quality control and harmonization following the FunGen-xQTL protocol^18^, with covariates including age at death, sex, post-mortem interval (when available), genetic principal components capturing ancestry, and hidden factors from molecular phenotype matrices. For qQTL calling, we tested associations at 19 equally spaced quantiles within topologically-associated domain boundaries (TADBs) or 2Mb cis-windows (whichever longer) for each gene using Qrank score test followed by hierarchical multiple testing correction at FDR < 0.05 to identify significant associations^19^. We complemented qQTL analysis with vQTL detection using QUAIL^20^ (Methods) to test associations with squared residuals from linear models, and iQTL analysis testing three interaction contexts: sex, *APOE* ε4 dosage, and estimated cell-type proportions in single-nucleus datasets, applying FDR thresholds of 0.25 for sex and *APOE* ε4 iQTLs and 0.05 for cell fraction iQTLs. For disease translation, we developed quantile-based transcriptome-wide association studies (qTWAS)^21^, which aggregates qQTL signals across quantiles to construct prediction models and test for associations with AD and aging-related traits, providing a complementary approach to standard TWAS that may capture genes whose pathogenic effects operate through distributional perturbations.

**Figure 1:**
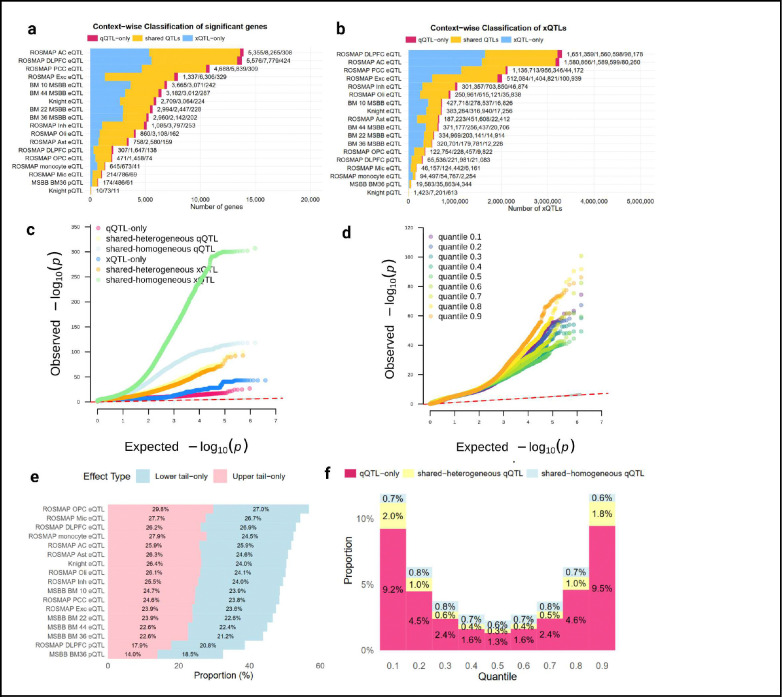
An atlas of molecular quantile QTL in human brain. **a-b**, Context-wise counts of significant gene–context associations (a) and variant–gene xQTL associations (b), stratified into qQTL-only (red), shared association detected by both qQTL and linear xQTL (gold), and xQTL-only (blue) across all datasets. **c**, Quantile–quantile (QQ) plots of observed versus expected −log_10_(*P*) values comparing different QTL classification groups: qQTL-only, shared-homogeneous qQTL, shared-heterogeneous qQTL, xQTL-only, shared-homogeneous xQTL, and shared-heterogeneous xQTL. **d**, QQ plots of observed versus expected −log_10_(*P*) values for qQTL at different quantiles τ=0.1,0.2,…,0.9. **e**, the proportion of qQTL with significant effects confined to lower quantiles (τ∈[0.05,0.3] versus upper quantiles (τ∈[0.7,0.95]). **f**, Quantile-specific effects by qQTL heterogeneity category. For each quantile τ=0.1,0.2,…,0.9, bars indicate the proportion of qQTL that are significant exclusively at that single quantile, stratified by qQTL-only, shared-heterogeneous qQTL, and shared-homogeneous qQTL.

Across all molecular contexts, we identified 9,744,370 significant qQTL associations involving 4,078,582 unique variant-gene pairs and 13,229 genes (Figure 1a,b). In comparison, vQTL analysis identified 1,713,820 significant associations, while iQTL analysis across the three interaction contexts yielded 5 sex-iQTLs, 139 *APOE* ε4-iQTLs, and 38,961 cell fraction-iQTLs. We integrated these non-linear QTL maps with AD and aging-related GWAS through stratified LD score regression to quantify heritability contributions, dissection of quantile-specific regulatory effects at established AD risk loci to reveal context-dependent mechanisms, and qTWAS to identify genes whose quantile-specific expression patterns associate with disease risk. The qTWAS prediction weights are publicly available at Synapse (synapse ID available upon publication) for application to external GWAS datasets, providing a resource for quantile-based transcriptome and proteome imputation across diverse complex traits.

### Quantile QTL landscapes reveal distributional regulatory effects beyond linear associations

To characterize the regulatory landscape captured by qQTL analysis, we compared qQTL discoveries with corresponding linear xQTL results at both variant and gene levels. We classified variant-gene pairs as “qQTL-only” if they achieved significance (FDR < 0.05) across quantiles (through Cauchy combination^22^ of quantile-specific p-values; Methods) but showed no significant association in linear regression (FDR > 0.05). Among variant-gene pairs significant in both analyses (“shared QTLs”), we further distinguished shared-homogeneous qQTLs, which showed consistent effect patterns across quantiles, from shared-heterogeneous qQTLs, which exhibited varying effect sizes across quantiles despite linear significance (see Methods for heterogeneity assessment using Chatterjee correlation test^23^). We collectively refer to qQTL-only and shared-heterogeneous associations as “heterogeneous qQTLs” (qQTL-het), representing regulatory effects characterized by distributional heterogeneity. At the gene level, we defined qQTL genes as genes harboring at least one significant qQTL association, and categorized them analogously based on overlap and heterogeneity patterns with xQTL genes.

Among the 13,229 qQTL genes identified, 435 (3.3%) were not detected as xQTL genes across contexts(Figure 1a). At the variant level, 5.7% qQTLs are qQTL-only (Figure 1b), and an additional 43% were shared-heterogeneous qQTLs, together comprising 48.7% qQTL-het (**Figure S1**). The proportion of qQTL-het varied across molecular contexts, with inhibitory neurons showing the highest proportion (52.88%, **Figure S1**).

To assess the relative statistical power of quantile versus linear regression, we examined p-value distributions across QTL categories (Figure 1c). Shared-homogeneous QTLs detected by linear regression showed the strongest associations, while shared-homogeneous QTLs detected by quantile regression had substantially larger p-values, indicating that quantile regression is less sensitive than linear regression for detecting pure mean effects and cannot fully replace linear analysis in such settings. However, for shared-heterogeneous QTLs, p-value distributions were comparable between methods, with quantile regression showing slightly stronger signals, demonstrating that when distributional heterogeneity is present, quantile regression leverages information across all quantiles and compensates for its reduced power in simpler mean-effect scenarios. QTLs detected uniquely by linear regression had moderately large p-values, representing signals missed by quantile regression due to limited power in mean-effect settings, though reassuringly this occurred only for weaker associations. QTLs detected uniquely by quantile regression (qQTL-only) showed the weakest signals overall, consistent with these representing subtler distributional effects operating at phenotypic extremes rather than mean-level changes.

Examining quantile-specific patterns revealed substantial enrichment at distributional extremes (Figure 1d,e). Comparing the number of significant qQTL-het across quantiles, we observed 6.9-fold and 7.0-fold enrichment, significant exclusively at τ = 0.1 and τ = 0.9, respectively, relative to the median quantile (τ = 0.5). Among shared-heterogeneous QTLs, 42.67% showed significantly stronger effects (**1.99**-fold change) at τ = 0.1 or τ = 0.9 compared to the median (Figure 1d), indicating that even for variants with detectable mean effects, the underlying regulatory architecture frequently involves distributional heterogeneity not fully captured by linear models. Notably, 13.46% of qQTL-het associations were significant exclusively at a single tail quantile (τ = 0.1 or τ = 0.9), potentially reflecting directional context-dependent effects (Figure 1e).

### Context-dependent regulatory architecture of quantile QTLs explains additional trait heritability

To assess the functional relevance of qQTL discoveries, we performed enrichment analysis using ~1,500 genomic and epigenomic annotations from multiple sources^24–28^ (Methods), testing whether qQTL variants showed preferential localization to regulatory elements compared to genome-wide background. We calculated enrichment odds ratios for each annotation category^29^, separately for qQTL-only and traditional xQTL variants, to isolate the contribution of distributional effects beyond mean-level associations and avoid confounding between the two.

**Figure 2:**
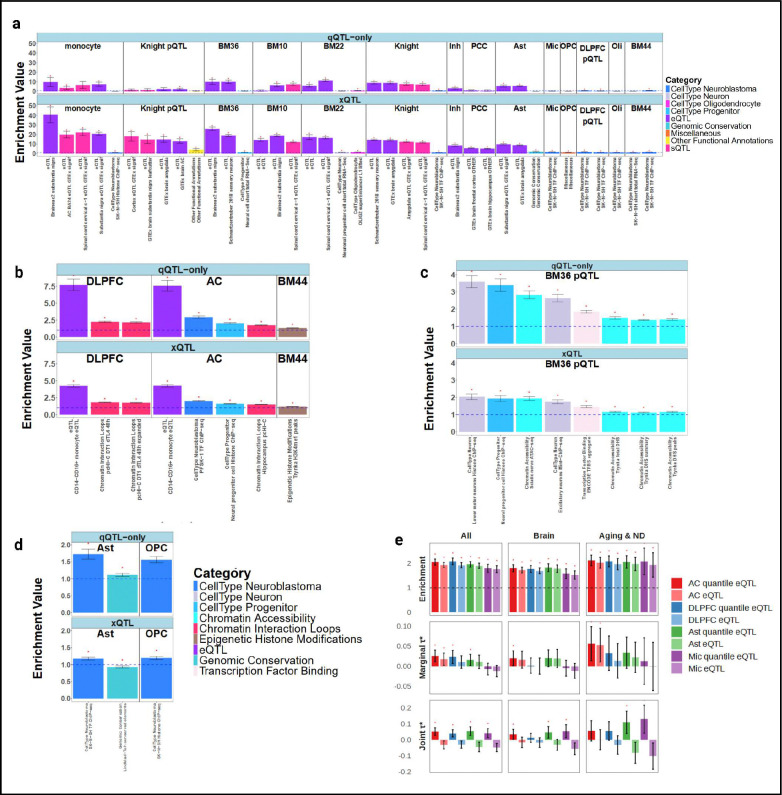
Functional characterization of quantile QTLs. **a–d**, EOO enrichment analysis. **a**, Functional annotations showing significantly higher enrichment for xQTL compared with qQTL-only variants. For each molecular context, the top five annotations with the largest enrichment differences (xQTL > qQTL-only) are displayed, with each category capped at two features per context. **b–d**, Functional annotations showing significantly higher enrichment for qQTL-only variants relative to xQTL, analyzed separately for bulk eQTL (b), protein QTL (c), and single-cell eQTL (d). **e**, sLDSC enrichment analysis. Comparison of qQTL versus xQTL stratified LD score regression enrichment across major brain regions (anterior cingulate cortex, dorsolateral prefrontal cortex) and brain cell types (astrocytes, microglia).

Comparing enrichment odds ratios identified annotation categories most differentially enriched between qQTL-only and xQTL variants (Figure 2a, **Table S2**). Categories showing stronger enrichment for xQTL variants were predominantly conventional eQTL and sQTL annotations, confirming that our linear xQTL analysis successfully recovers known transcriptional regulation mechanisms. In contrast, qQTL-only variants showed top enrichments in several annotation categories that provide insights into non-linear regulatory mechanisms, with patterns varying across molecular contexts.

For bulk tissue eQTLs, qQTL-only variants showed the strongest enrichment in cell-type-specific eQTL annotations (7.71-fold, p = 1.06 × 10^−18^, Figure 2b), which likely reflects the fact that when genetic effects are specific to one cell type but absent in others, they manifest as non-linear associations in bulk tissue measurements depending on the fraction of the cell type (cell fraction interaction effects). This interpretation was further supported by enrichment in cell-type-specific regulatory elements (1.35-fold, p = 3.90 × 10^−402^, Figure 2b), which serve as proxies for cell-type-specific genetic effects. Notably, qQTL-only variants also showed substantial enrichment in chromatin interaction loops (**2.25**-fold, p = 6.85 × 10^−132^, Figure 2b). This pattern is consistent with a “two-hit” model of regulatory variation, where genetic effects are contingent on chromatin state: the regulatory element must first be in an open, accessible configuration or engaged in active three-dimensional contacts before genetic variants can exert their effects, creating gene-by-environment interactions at the chromatin level. For protein QTLs, qQTL-only variants also showed enrichment in chromatin accessibility regions (2.83-fold, p = 1.12 × 10^−36^) and transcription factor binding sites (1.86-fold, p = 8.45 × 10^−147^, Figure 2c); one possible explanation is that these genomic regions, when transcribed, may harbor RNA-binding protein recognition motifs that regulate post-transcriptional processes in a context-dependent manner, though this pattern warrants further investigation.

For cell-type-specific analyses, qQTL-only variants in single-nucleus eQTLs were most strongly enriched in regulatory annotations from other cell types rather than the matched cell type (for astrocytes using TF ChIP-seq annotations: 1.73-fold, p=1.41×10^−31^ in neuroblastoma versus 1.08-fold, p=8.85×10^−13^ in astrocytes; Figure 2d), suggesting that genetic effects in one cell type may depend on the regulatory state or signaling from other cell types, potentially reflecting cell-cell communication mechanisms or trans-cellular regulatory influences in the brain.

To quantify the contribution of qQTL variants to complex trait heritability, we applied stratified LD score regression (sLDSC)^27,28^ using summary statistics from 86 GWAS, including 49 brain-related traits, within which 13 were aging or neurodegenerative traits (1 of which Alzheimer’s disease GWAS) (Methods). We constructed binary annotations for qQTL and xQTL variants, and estimated the proportion of trait heritability explained by variants in each annotation while controlling for a baseline model of 97 functional categories (Methods). In marginal analyses, qQTL annotations showed slightly higher heritability enrichment than xQTL annotations across multiple tissue and cell type contexts (Figure 2e). To assess the independent contribution of each annotation, we performed joint modeling including both qQTL and xQTL annotations in sLDSC. We found that when conditioning on xQTL, qQTL still explained substantial additional heritability (Figure 2e), demonstrating that distributional genetic effects captured by qQTL contribute trait heritability beyond mean-level associations in conventional xQTL.

### Quantile QTL reveals complex context-dependent effects at AD risk loci missed by linear models

To illustrate the biological insights enabled by qQTL analysis, we examined regulatory effects at candidate AD risk genes where distributional genetic effects were detected but linear associations were weak or absent, including *PITRM1*, a simple variance QTL completely missed by linear analysis and *TMEM106B*, exhibiting lower-quantile-specific effects in excitatory neuron combined with interaction effects. We also examine the *CHRNE*/*SCIMP*/*RABEP1* gene cluster which shows complex regulatory architecture involving multiple loci with non-linear effects.

### *PITRM1*: A variance QTL undetected by linear regression

**Figure 3:**
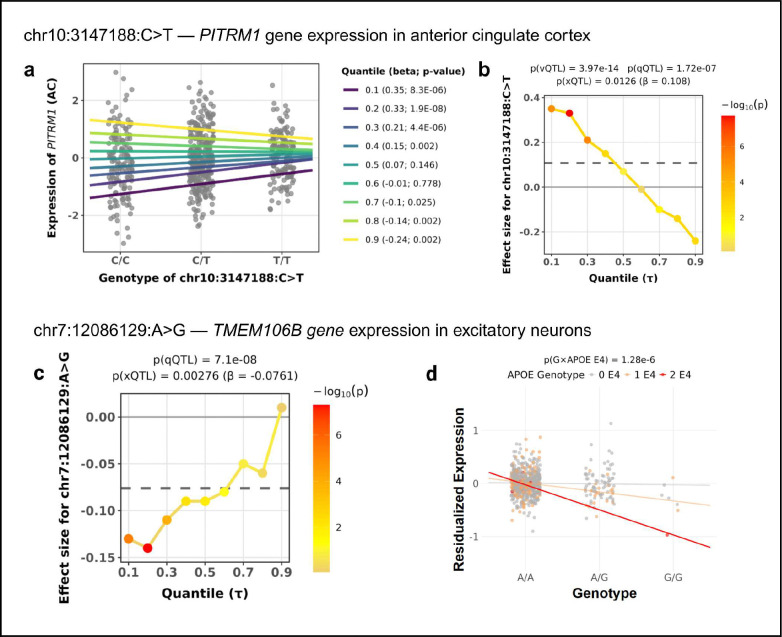
Examples of qQTL effects in human brain tissue. **a–b**, *PITRM1* expression in anterior cingulate cortex for variant chr10:3147188:C>T. **a**, Quantile regression fits across quantiles (τ=0.1-0.9) stratified by genotype, showing differences in expression patterns across the distribution. **b**, Quantile regression coefficient profiles illustrating variation in effect sizes across τ. **c–d**, *TMEM106B* expression in excitatory neurons for variant chr7:12086129:A>G. **c**, Quantile regression coefficient profiles across τ. **d**, Interaction plot of the genotype with APOE-ε4 genotype.

*PITRM1* (pitrilysin metallopeptidase 1) encodes a mitochondrial matrix protease implicated in mitochondrial dysfunction and has been associated with AD pathology^30^. In ROSMAP^17^ anterior cingulate cortex (AC), the variant chr10:3147188:C>T showed no significant association with *PITRM1* expression in linear analysis (β = 0.108, p = 0.0126) but is a strong vQTL (p = 3.97 × 10^−14^, Figure 3a) and qQTL (p = 1.72 × 10^−7^, Figure 3b). Quantile analysis suggested significant effects towards both tail quantiles, with minimal effect at the median. The quantile effect sizes exhibited symmetric patterns at the lower and upper tails with opposite signs, consistent with a variance-increasing effect without shifting the mean (Figure 3b). Despite its potential role in AD, this variant does not exhibit an AD GWAS association (minimum p-value > 0.01 across AD data-sets).

### *TMEM106B*: Dissecting multiple non-linear regulatory effects at qQTL loci

*TMEM106B* (transmembrane protein 106B) is a lysosomal membrane protein whose expression in neurons regulates lysosomal function, pH, and trafficking, with reduced neuronal TMEM106B expression observed in AD brains and implicated in disease pathogenesis^31^. In ROSMAP single-nucleus excitatory neurons, the variant chr7:12086129:A>G showed significant qQTL association specifically at the lower quantile (τ = 0.1: β = −0.13, q = 0.04; τ = 0.15: β = −0.13, q = 1.30 × 10^−6^; τ = 0.2: β = −0.14, q = 1.44 × 10^−4^) with progressively weaker effects toward higher quantiles (τ = 0.5: β =−0.09, q = 0.175; τ = 0.9: β = 0.01, q = 0.962, Figure 3c). The negative effect sizes at lower quantiles suggest the risk allele further reduces *TMEM106B* expression in the low-expressing subpopulation, potentially exacerbating lysosomal dysfunction in these vulnerable neuronal states. Linear regression detected a modest mean effect (β = −0.076, p = 2.76 × 10^−3^). This variant showed only moderate association with AD risk in GWAS (p = 1.9 × 10^−3^).

To dissect potential mechanisms underlying this quantile-specific effect, we tested for interaction with *APOE* ε4 carrier status. We observed strong evidence *APOE* ε4 interaction (interaction β = −0.239, p = 1.26 × 10^−6^, q = 4.86 × 10^−3^), with negligible mean genetic effects in ε4 non-carriers (main effect p = 0.682) but progressively stronger suppression in ε4 heterozygotes and homozygotes (Figure 3d), suggesting that the variant exacerbates *TMEM106B* deficiency specifically in the *APOE* ε4 background, which itself confers AD risk. Both iQTL and qQTL reveal that the variant targets already vulnerable populations (ε4 carriers and low-expression individuals, respectively) to further exacerbate risk. The iQTL, by explicitly identifying *APOE* ε4 as the interacting factor, provides a possible explanation for the qQTL: if the variant acts primarily in ε4 carriers, and ε4 carriers are enriched at lower expression levels (as both ε4 status and low *TMEM106B* expression associate with elevated AD risk), then the genetic effect would concentrate at lower quantiles, producing the observed quantile-specific pattern. Testing for cell fraction interactions with six major cell types revealed no significant associations, ruling out cellular composition as an additional contributor to qQTL.

### *CHRNE/SCIMP/RABEP1*: A gene cluster with complex non-linear regulation

The chromosomal region harboring *CHRNE* (cholinergic receptor nicotinic epsilon subunit)^32^, *SCIMP* (SLP adaptor and CSK interacting membrane protein)^33^, and *RABEP1* (rabaptin, RAB GTPase binding effector protein 1)^34^ contains multiple AD-associated variants in moderate linkage disequilibrium, but the causal gene targets remain unresolved. Using colocalization analysis between FunGen-xQTL data and AD GWAS, we identified significant eQTL associations for *SCIMP* in microglia, astrocytes, excitatory neurons, inhibitory neurons, and relevant bulk tissues; *RABEP1* in microglia, oligodendrocytes, and bulk tissues; and *CHRNE* in bulk tissues (**Table S3**).

**Figure 4:**
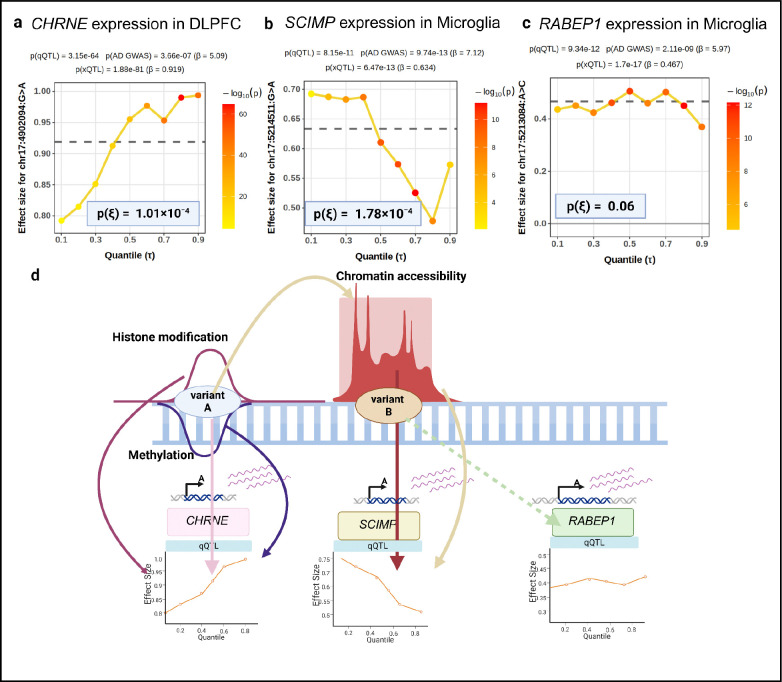
Examples of *CHRNE/SCIMP/RABEP1*: qQTL patterns and regulatory annotations in human brain. **a,** *CHRNE* expression in DLPFC. Quantile regression (qQTL) estimated effect sizes for variant chr17:4902904:G>A across quantiles (τ=0.1-0.9). **b,** *SCIMP* expression in microglia. qQTL estimated effect sizes for variant chr17:5214511:G>A across quantiles (τ=0.1-0.9). **c,** *RABEP1* expression in microglia. qQTL estimated effect sizes for variant chr17:5213084:A>C across quantiles (τ=0.1-0.9). **d,** Illustrative schematic of potential regulatory features in the locus. The panel summarizes putative regulatory elements, epigenomic marks (chromatin accessibility, histone modification, and DNA methylation), variant positions, and associated qQTL effect-size profiles for *CHRNE*, *SCIMP*, and *RABEP1*.

We then assessed whether these eQTLs exhibited quantile-specific effects (qQTL-het). For *SCIMP* in microglia, the AD risk variant chr17:5214511:G>A showed significant qQTL with stronger positive effects at lower quantiles than higher quantiles (Figure 4). For *CHRNE* in brain bulk tissue, qQTL at chr17:4902094:G>A showed stronger effects at higher quantiles than lower quantiles (Figure 4a **in some bulk**), opposite to the *SCIMP* pattern (Figure 4b **across different cell types**). *RABEP1* exhibited simple linear associations across all contexts with no qQTL-het identified (Figure 4c **across cell type nothing**).

To characterize these quantile-specific patterns, we integrated epigenomic data including snATAC-seq^35^, histone modification (H3K9ac), and DNA methylation^36^ from FunGen-xQTL project (Methods). We identified chromatin accessibility peaks at the chr17:5214511 locus overlapping *SCIMP* that showed cell-type-specific variation (Figure 4c **plot raw data**), and H3K9ac and methylation marks (Figure 4d **plot raw data**) at the chr17:4902094 locus overlapping *CHRNE* regulatory elements. *RABEP1* appeared to lack corresponding epigenomic marks at either position in our datasets. Epigenetic QTL mapping using fSuSiE^37^ and mfSuSiE (Methods) revealed that chromatin accessibility, H3K9ac, and methylation were all associated with the locus harboring chr17:4902094:G>A (Figure 4e plot effects for caQTL, Figure 4f **plot effects of mQTL and haQTL**).

For *CHRNE*, mediation analysis indicated the H3K9ac and methylation marks do not mediate the expression effects (mediation proportion < 2%, p = 0.70), suggesting that variants in locus around chr17:4902094 simultaneously reduce DNA methylation, increase H3K9ac (both promoting transcription), and act as an eQTL directly. In samples with already-high *CHRNE* expression, these mechanisms may compound to drive expression higher, resulting in larger effects at upper quantiles. For *SCIMP*, the regulatory architecture involves coordination between two loci in modest LD (r^2^ = 0.12): variants around chr17:4902094 influences chromatin accessibility at the distal *SCIMP* regulatory region, while variants around chr17:5214511 reside within that region and directly regulate *SCIMP* expression. The larger effects at lower quantiles for chr17:5214511:G>A suggest the variant either compensates for partial chromatin accessibility or shows reduced impact when chromatin is fully open when available transcriptional regulators such as RNA polymerase and TF are already maximally engaged (Figure 4f **eventually connecting all of the above from a to e except for *RABEP1***).

These findings illustrate how qQTL analysis reveals regulatory complexity at disease-associated loci and guides deeper investigation. While standard eQTL analysis implicates all three genes at this locus, qQTL analysis distinguishes *SCIMP* and *CHRNE* as having context-dependent regulation from *RABEP1* with uniform linear effects. Whether this complexity represents therapeutic advantage or challenge remains unclear: multi-modal regulation may offer multiple intervention points, but could also require coordinated targeting of multiple mechanisms to achieve full efficacy.

### Quantile QTL captures variance effects and complements interaction QTL discovery

**Figure 5:**
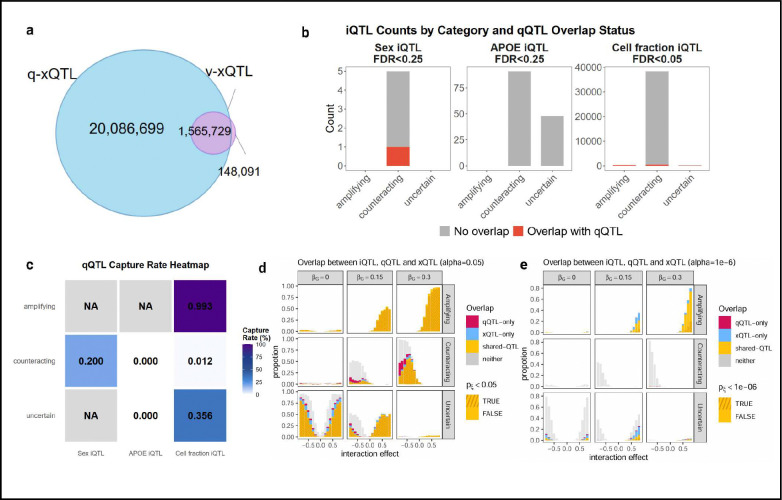
Connection of non-linear QTL to quantile QTL. **a**, Overlap between vQTL and qQTL at q-value < 0.01. **b**, Counts of iQTL classified into amplifying, counteracting, and uncertain categories (methods) across sex, APOE ε4, and cell-fraction interaction contexts. Bars indicate whether each iQTL overlaps a qQTL (red) or not (grey). **c**, qQTL capture rates for each iQTL category across interaction contexts. The heatmap reports the proportion of iQTLs in each category recovered by qQTL analysis. **d**, Simulation-based comparison of iQTL, q-xQTL, and linear xQTL detection at significance threshold α = 0.05. Each panel reports the proportion of findings classified as qQTL-only, xQTL-only, shared-QTL, or neither. For shared-QTLs, quantile heterogeneity is defined as $p_{\xi }<0.05$ ; heterogeneous shared-QTLs are highlighted with yellow hatching. **e**, Same simulation framework as in d, evaluated at genome-wide threshold α = 1×10^−6^. For shared-QTLs, quantile heterogeneity is defined as $p_{\xi }<1\times 10^{-6}$;

To contextualize qQTL findings relative to established approaches for non-linear genetic effects, we systematically compared discoveries across vQTL and iQTL analyses performed on the same datasets. Of the 1,713,820 significant vQTL (q-value<0.01) associations identified, 91.36% were also detected as qQTLs, predominantly showing significance at both tail quantiles (τ = 0.1 and τ = 0.9) consistent with variance-increasing effects. Conversely, most qQTLs were not detected as vQTLs (Figure 5a), supporting vQTL as a special case of qQTL where genetic effects increase variability symmetrically across the distribution.

Following existing iQTL analysis practice, we categorized effects as amplifying (interaction and main effects have the same sign), counteracting (opposite signs), or uncertain (no significant main effect) based on stratified genetic effects^4^ (Methods). Using established lenient thresholds (FDR < 0.25 for sex and *APOE* ε4, FDR < 0.05 for cell fraction), we detected 5 sex-iQTLs, 139 *APOE* ε4-iQTLs, and 38,961 cell fraction-iQTLs as previously described, substantially fewer compared to qQTL. The majority were counteracting or uncertain (Figure 5b), with counteracting substantially outnumbering amplifying, i.e., interaction factors tend to weaken rather than strengthen main genetic effects — for cell fraction interactions, this may reflect compensatory regulation where increased cell abundance allows individual cells to regulate less strongly while maintaining the same aggregate expression.

The overlap between iQTL and qQTL/xQTL varied by interaction type: qQTLs recovered most amplifying iQTLs (99.3% for cell fraction). In contrast, counteracting and uncertain iQTLs showed minimal overlap with qQTL (≤20% and ≤35.6%, respectively; Figure 5c). When applying stringent FDR < 0.05 across all interaction contexts with additional QC filtering (Methods), only 2 sex and 4 *APOE* ε4 iQTLs remained, nearly all counteracting or uncertain (**Table S4**). None were captured by qQTL, and few replicated at nominal significance in independent datasets from the same cohort, suggesting limited robustness of these interaction signals.

To understand the relationship between iQTL directionality and qQTL detection, we performed simulations modeling genetic main and interaction effects calibrated to real data parameters, analyzing each as iQTL, qQTL, and linear xQTL (Methods). Under well-powered condition (by setting α = 0.05), amplifying iQTLs showed >99% overlap between qQTL and xQTL when interaction effects were strong (Figure 5d). In contrast, counteracting iQTLs were substantially better detected by qQTL than xQTL (Figure 5d), as opposing subgroup effects cancel in the mean but remain visible at distributional quantiles. Uncertain iQTLs were poorly detected by both. Notably, most (95%) amplifying iQTLs detected by qQTL exhibited evidence of quantile heterogeneity (Chatterjee correlation p_ξ_<0.05). Under more realistic stringency for type I error (α = 1×10^−6^), detection rates dropped substantially (Figure 5e). For counteracting interactions, qQTL remained slightly more powerful than xQTL but absolute detection was minimal for both approaches. Even for amplifying interactions where both approaches maintain reasonable power, only 22% showed significant quantile heterogeneity at genome-wide threshold (p_ξ_< 1 × 10^−6^). These simulation results match our empirical observations: qQTL offers better sensitivity than xQTL for counteracting interactions and, when capturing either interaction type, reveals distributional heterogeneity that can guide targeted interaction testing. As we demonstrated with *TMEM106B*, qQTL signals can prioritize specific loci and candidate interaction factors for formal iQTL follow-up, as a practical strategy when comprehensive iQTL testing across many factors is computationally expensive yet yields few discoveries due to limited statistical power. However, our simulations and empirical comparisons demonstrate that iQTL and qQTL are complementary approaches, and caution must be taken when adopting such qQTL-guided approach as many true iQTLs lack detectable distributional signatures and would be missed by qQTL screening.

Among the 4 *APOE* ε4 and 2 sex iQTLs passing stringent FDR < 0.05 — predominantly counteracting or uncertain, and missed by qQTL — we examined replication across independent datasets. For *APOE* ε4 interactions, only *TTN* in excitatory neurons replicated at nominal significance (p < 0.05) in a second study (Methods; **Table S5**). However, closer examination revealed the signal is likely driven by outliers in homozygous alternative allele carriers (**Figure S2**). Finally, we applied a candidate gene approach to AD GWAS loci, using relaxed per-gene thresholds (interaction q < 0.05) with Bonferroni correction across loci (5×10^−4^). This identified 12 genes with cell fraction iQTLs (**Table S5**). However, closer examination revealed these interactions were not robust: effects were small, genotype patterns violated additive model assumptions with non-progressive dose-response (e.g., 0→2→1 rather than 0→1→2 for allele dosage), or associations appeared outlier-driven (**Figure S2**). Even with focused hypothesis-driven approaches, we found few robust iQTLs for AD genes across the factors we systematically tested.

### Distributional genetic effects reveal additional gene associations for Alzheimer’s disease and telomere length

To assess the disease relevance of distributional genetic effects and provide a translational resource for GWAS interpretation, we developed quantile-based transcriptome-wide association studies (qTWAS), which integrates quantile-specific prediction models to capture how genetic variation influences the entire expression distribution^21^ (Methods). Unlike previous sections focused on qQTL statistical significance, qTWAS leverages prediction accuracy regardless of whether variants show significant qQTL effects, testing whether distributional modeling improves gene-trait association detection compared to mean-based approaches. We built qTWAS weights for ~70,000 gene-context combinations across 18 molecular contexts and applied them to four AD GWAS datasets^38–40^ and one telomere length GWAS^41^ (average n≈500,000), using Bonferroni-corrected p < 0.05 per context as the significance threshold. For comparison, we extracted standard TWAS weights from FunGen-xQTL across the same contexts, where eight TWAS methods were employed to optimize detection of mean-level genetic effects.

Across the four AD GWAS datasets, qTWAS identified 347 significant gene-context associations representing 184 unique genes, including 64 genes not significant by standard TWAS (qTWAS-only genes) (Figure 5a,b, **Table 2**). For telomere length, qTWAS identified 1,705 significant associations with 688 genes, including 90 qTWAS-only genes (Figure 5c,d, **Table 2**). qTWAS identified 39% as many genes as standard TWAS, with most being TWAS-shared, as expected given years of methodological optimization of high-dimensional regression approaches for TWAS and greater power of linear xQTL over qQTL. However, qTWAS-only genes remain substantial: For Alzheimer’s disease, 35% of qTWAS genes were unique at the gene level, and 43% of gene–context associations were qTWAS-only. For telomere length, 13% of qTWAS genes and 23% of gene–context associations were qTWAS-only (Figure 5a-d), indicating some genes show qTWAS-only signals in specific contexts despite being TWAS-significant in others, demonstrating context-specificity of distributional effects. To assess whether qTWAS detects robust signals, we examined overlap with suggestive TWAS associations (p < 1×10^−4^). At the gene level, 95% of AD qTWAS genes showed suggestive TWAS signals, while 87% of telomere length qTWAS genes did (Figure 5e,f), indicating qTWAS captures genes with distributional genetic effects strong enough to produce suggestive mean-level signals but not significant under linear modeling. At the gene-context level, an even smaller proportion of telomere length qTWAS gene-context associations had suggestive TWAS signals compared to AD (45% vs 70%, Figure 5g), suggesting telomere length associations may involve more non-linear regulatory effects than AD.

To assess novelty of qTWAS-only genes relative to other FunGen-xQTL evidence, we compared them against multi-context colocalization^42^, multi-trait fine-mapping^43^, and complete TWAS results across 62 total xQTL contexts and datasets. Only 25% of qTWAS-only genes were detected by these complementary approaches, indicating most represent new discoveries enabled by distributional genetic modeling (Figure 5h). To biologically validate these new discoveries, we performed pathway enrichment analysis^44,45^ for AD and telomere length genes. For AD, qTWAS genes showed stronger enrichment in immune-related biological processes, including macrophage proliferation, NK cell cytotoxicity and regulation (Figure 5i). Cellular component enrichments highlighted lipid metabolism pathways (HDL particle, protein-lipid complex, VLDL particle, TG-rich lipoprotein, phospholipid efflux), and molecular function analysis revealed tau binding — all established AD mechanisms. For telomere length, qTWAS-only genes showed enrichment in telomerase RNA localization to Cajal bodies (**Figure S3**). Analyzing all qTWAS genes revealed stronger enrichment in immune-related pathways spanning autoimmune diseases (Type I diabetes, allograft rejection, GVHD, autoimmune thyroid, asthma), antigen presentation mechanisms (MHC class I and II pathways), telomere maintenance components (telomere cap complexes, chromosomal telomeric repeats), and peptide binding functions (Figure 5j). These enrichment patterns suggest qTWAS captures genes whose pathogenic effects may operate through threshold-dependent or context-specific mechanisms rather than through simple linear dose-dependent relationships: for example immune dysfunction in both neurodegeneration and aging where function deteriorates at expression thresholds, and telomere maintenance processes where critical length thresholds trigger cellular responses.

## DISCUSSION

Traditional statistical genetics methods quantify whether genetic variants affect molecular phenotypes and estimate effect direction and magnitude on the mean. However, this average behavior may obscure regulatory complexity relevant to disease mechanisms. Our comprehensive analysis across 28 molecular contexts in the aging human brain provides a non-linear QTL resource encompassing qQTL, vQTL, and iQTL, demonstrating that quantile regression reveals how genetic effects vary across the expression distribution, capturing context-dependent regulation missed by linear models. qQTL variants, while less powerful than linear xQTL due to current methodological constraints, explained additional disease heritability beyond contributions from linear xQTL. These variants showed distinct functional architecture: enrichment in cell-type-specific regulatory elements in bulk tissues, enrichment in cross-cell-type regulatory elements in single-nucleus data suggesting cell-cell interactions, and enrichment in chromatin accessibility regions and long-range chromosomal contacts consistent with two-hit regulatory mechanisms. By definition, qQTL makes vQTL a special case of symmetric distributional effects, which we confirmed empirically. Although not replacing iQTL, qQTL serves as a screening tool for heterogeneous genetic effects that can prompt targeted interaction analysis.

Detailed investigations of molecular QTL regulation at AD risk loci illustrated diverse qQTL patterns, with enrichment at distributional extremes (τ = 0.1 and 0.9) suggesting genetic effects operating under specific biological conditions, and revealed what standard approaches miss: linear regression failed to detect *PITRM1*, which exhibited symmetric variance effects where certain genotypes show greater phenotypic plasticity without clear directional impact. vQTL cannot capture asymmetric distributional patterns exemplified by *TMEM106B* (operating at one quantile extreme) and *CHRNE*/*SCIMP*/*RABEP1* (showing progressive effect changes across quantiles with coordinated regulation involving DNA methylation, H3K9ac, and chromatin accessibility). For *TMEM106B*, one locus showed lower-quantile-specific effects with *APOE* ε4 interaction partially explaining this pattern, while another locus showed both linear and quantile effects, illustrating how qQTL can prompt targeted interaction testing. However, iQTL detection in brain showed limited power and robustness consistent with prior studies. While iQTL may be informative for some genes, our systematic testing across established AD loci revealed few robust interactions with sex, *APOE* ε4, or cell fraction, with many signals driven by outliers and poor replication, making comprehensive iQTL testing computationally prohibitive and targeted hypothesis-driven approaches more practical.

Several limitations warrant consideration and highlight possible directions for future work. First, qQTL methodology remains underdeveloped relative to linear QTL frameworks. Current implementations (based on QRank method) show lower power for detecting pure mean effects despite theoretical expectations that xQTL and vQTL should emerge as special cases of qQTL. Additionally critical methodological gaps include fine-mapping approaches that leverage quantile information to identify causal variants, sophisticated high-dimensional regression models for prediction (our simple qTWAS models partially explain fewer discoveries than state-of-the-art TWAS methods), and causal inference extensions of Mendelian randomization and causal TWAS to quantile regression. Developing these tools and systematically applying fine-mapping with colocalization to qQTL associations remain priorities for enabling variant-level functional follow-up. Second, while qQTL shows better power for capturing certain interactions where linear regression fails, our simulations demonstrate it still misses many interaction signals under stringent FDR correction. Although following up qQTL-prioritized loci with targeted iQTL testing of specific hypothesized interaction factors proved productive in our *TMEM106B* case study, we caution that qQTL cannot fully replace comprehensive iQTL screening. Third, dissecting qQTL signals requires integrating targeted iQTL analysis, functional annotations, and complementary multi-omics data, as demonstrated in our AD risk gene case studies. While increasingly feasible with expanding molecular datasets such as our FunGen-xQTL project, such integrative interpretation remains resource-intensive for individual researchers investigating specific genes. To address this, we are working as a consortium to provide comprehensive integrative analyses for AD GWAS loci, connecting multi-scale molecular evidence including qQTL signals. Similar community efforts to create prioritized, modular multi-omics resources centered on GWAS signals would greatly facilitate functional interpretation across complex diseases.

Beyond statistical significance, the primary value of qQTL lies in revealing effect patterns across the phenotype distribution that inform biological mechanisms where quantile-specific effects point to context-dependent regulatory complexity. However, for researchers focused solely on discovery power, qQTL currently provides modest gains in significant associations relative to the computational investment for genome-wide applications — reflecting current methodological limitations rather than inherent constraints of the approach. Nevertheless, we provide efficient computational protocols and pipelines (see Code Availability) that streamline and automate large-scale qQTL analysis, integrating both qQTL discovery and qTWAS weight training for those seeking to build prediction models. Researchers can adopt qQTL either as a genome-wide discovery tool for comprehensive regulatory effects characterization or as a targeted follow-up approach for specific loci of interest (GWAS risk loci, candidate genes) to characterize regulatory complexity beyond mean effects. For disease gene prioritization, we provide a pretrained qTWAS resource for human brain across 22 molecular contexts, offering an immediately accessible database requiring only downloading prediction weights to apply quantile-based transcriptome and proteome imputation to GWAS data, potentially uncovering gene associations missed by standard TWAS.

In summary, qQTL analysis provides a complementary dimension to standard molecular QTL mapping, capturing context-dependent regulatory effects missed by linear models and offering additional evidence for disease gene prioritization. We provide a comprehensive non-linear QTL atlas and qTWAS resource for the human brain that enables characterization of distributional genetic effects across complex traits, with potential to inform experimental validation and therapeutic target identification.

## METHODS

https://www.overleaf.com/project/652803dda9318d04d5f46393

## METHODS

### Pipelines for molecular QTL identification

Consider a sample with n independent individuals, indexed by i=1,⋯,n. Let $Y_{i}$ denote the phenotype value of a quantitative trait for individual i, such as gene expression level for a gene of interest. Let m be the number of the generic variants in a gene region. Denote $G_{ij}$ for j=1,⋯,m as genotype of genetic variant j for individual i, coded additively as the number of minor alleles $\left(G_{ij}\in \{0,\;1,\;2\}\right)$. Let $C_{i}={\left(C_{i1},\cdots ,C_{iq}\right)}^{⊤}$ be a vector of q covariates for individual i. Let $\theta_{j}$ denote a parameter quantifing the genetic effect of variant j on the feature of interest under the conditional distribution $F_{Y_{i}|G_{ij},C_{i}}$. The specific feature depends on the type of quantitative trait locus (QTL): the conditional mean for linear QTL (xQTL), conditional quantiles for quantile QTL (qQTL), conditional variantce for variance QTL (vQTL), and genotype-by-interacting factor for interaction QTL (iQTL), etc. To test whether variant j is associated with the feature of interest, we consider the null hypothesis $H_{0}:\theta_{j}=0$. Detailed model specifications are provided in **Supplementary Note S.1**.

For *cis*-QTL identification, we restrict testing to variants within topologically-associated domain boundaries (TAD-Bs) or 2Mb *cis*-windows (whichever longer) for each gene^1^. We develop a pipeline comprising quality control and hierarchical multiple-testing correction^2^.

#### Quality control.

Genetic variants are filtered based on minor allele frequency (MAF≥0.01) and minor allele count (MAC≥5), retaining variants meeting either criterion to ensure robust statistical inference. Missing genotypes are imputed using mean values, and variants with zero variance were excluded^3^.

#### Multiple-testing correction.

We apply a two-stage hierarchical procedure following Huang et al.^4^. (1) Stage 1: claim the significant genes. At the gene-level, we denote the Bonferroni-adjusted p-value for variant j within the cis-window of one gene as ${\tilde{p}}_{g,j}$. Specifically, we denote ${\tilde{p}}_{g}^{*}$ as the Bonferroni-adjusted p-value of its lead cis-variant. Then, the Benjamini–Hochberg procedure is applied on ${\tilde{p}}_{g}^{*}$ across all genes to control the false discovery rate (FDR) at 5%. (2) Stage 2: claim the significant QTL based on significant genes. For genes passing the FDR threshold from stage 1, we define a data-driven variant-level significance threshold. The datadriven threshold is defined as $\tau =max\;{\tilde{p}}_{g}^{*}$ across all significant genes. Therefore, a variant-gene pair is finally declared significant if ${\tilde{p}}_{g,j}<\tau$. Based on this two-stage hierarchical multiple testing correction, gene-level FDR is controlled at 5% and variant-level discoveries are restricted to significant genes with a Bonferroni threshold τ (see details in **Supplementary Note S.2**).

### Quantile rank score test to identify quantile QTL

To detect qQTL with distributional-wise effects across the gene expression distribution and may be missed by conventional QTL identifications, we use a computationally efficient quantile rank score test^5^ and aggregate evidence across multiple quantile levels using the Cauchy combination method^6^.

For a given quantile level τ∈(0,1), denote the τth quantile of $Y_{i}$ as $Q_{Y_{i}}(\tau )$. We model the relationship between $Q_{Y_{i}}(\tau )$ and genetic variant $G_{ij}$ conditional on covariates $C_{i}$ through a linear quantile regression model^7^:

(1) $$Q_{Y_{i}}\left(\tau |G_{ij},C_{i}\right)=\alpha_{0}(\tau )+C_{i}^{⊤}\alpha (\tau )+G_{ij}\beta_{j}(\tau ),$$

where $\alpha_{0}(\tau )$, α(τ), and $\beta_{j}(\tau )$ are quantile-specific intercept and coefficients. The test statistic of rank score test is given by

(2) $$S_{j,\tau }=n^{-1/2}\sum_{i=1}^{n}\;G_{ij}^{*}\phi_{\tau }\left\{Y_{i}-C_{i}^{⊤}\hat{\alpha }(\tau )-{\hat{\alpha }}_{0}(\tau )\right\},$$

where $\phi_{\tau }(u)=\tau -1(u<0)$, $C_{i}$ are the columns of matrix C, and $G_{j}^{*}=(I-C{\left(C^{⊤}C\right)}^{-1}C^{⊤})G_{j}$ represents the projection of $G_{j}$ on the orthogonal complement of the column space of $C,\;C={\left(C_{1}^{⊤},\cdots ,C_{n}^{⊤}\right)}^{⊤}$, $G_{j}={\left(G_{1j},\cdots ,G_{nj}\right)}^{⊤}$, $G_{j}^{*}={\left(G_{1j}^{*},\cdots ,G_{nj}^{*}\right)}^{⊤}$. Here, ${\hat{\alpha }}_{0}(\tau )$ and $\hat{\alpha }(\tau )$ are estimated under $H_{0}$, requiring the null model to be fitted only once for all variants. Under the null hypothesis $H_{0}$, the standardized statistic $S_{j,\tau }/\sigma_{j,\tau }∼N(0,\;1)$, where $\sigma_{j,\tau }^{2}=\tau (1-\tau )G_{j}^{*⊤}G_{j}^{*}/n$. We denote $p_{\tau }$ as the corresponding p-value for a given quantile-level τ. To aggregate evidence across multiple quantiles, we apply the Cauchy combination method^6^ and claim significant qQTL using the two-stage hierarchical multiple-testing correction described above; see details in Pipelines for molecular QTL identification. These significant qQTL are subsequently used to construct quantile-specific prediction weights in quantile TWAS (qTWAS) framework, as described below.

### Quantile TWAS to identify distribution-specific disease associations

To identify genes whose quantile-specific regulatory patterns contribute to disease risk, we consider a qTWAS framework that integrates qQTL-derived prediction weights with GWAS summary statistics for a disease of interest. This approach extends traditional TWAS by capturing disease associations driven by genetic regulation at specific portions of the gene expression distribution rather than mean expression levels alone. All subsequent steps are performed for each gene independently, for notational simplicity, we suppress the gene index g.

#### Quantile weight estimation.

Significant qQTLs are harmonized with external Alzheimer’s Disease Sequencing Project (ADSP) linkage disequilibrium (LD) reference panels, further removing highly correlated signals with an $R^{2}$ threshold of 0.8. Let ${\left\{G_{\text{final},j}\right\}}_{j=1}^{p}$ be the final variant set of qQTL for the resulting filtered variant set, we estimate quantile-specific genetic effects by fitting a joint quantile regression model across a fine grid of 99 equidistant quantile levels (τ∈{0.01,0.02,…,0.99}),

(3) $$Q_{Y_{i}}\left(\tau |G_{\text{final},i}^{⊤},C_{i}\right)=\gamma_{0}(\tau )+C_{i}^{⊤}\gamma (\tau )+\sum_{j=1}^{p}\;G_{\text{final},ij}\omega_{j}(\tau ),$$

where $\gamma_{0}(\tau )$, γ(τ), and ${\left\{\omega_{j}(\tau )\right\}}_{j=1}^{p}$ are quantile-specific intercept, covariate effects, and genetic effect coefficients, respectively. These parameters were estimated by minimizing the quantile check loss function,

(4) $$\left({\hat{\gamma }}_{0}(\tau ),\hat{\gamma }(\tau ),\hat{\omega }(\tau )\right)=\underset{\gamma_{0}(\tau ),\gamma (\tau ),\omega (\tau )}{argmin}n^{-1}\sum_{i=1}^{n}\;\rho_{\tau }\left\{Y_{i}-G_{\text{final},i}^{⊤}\omega (\tau )-C_{i}^{⊤}\gamma (\tau )-\gamma_{0}(\tau )\right\}.$$

The resulting quantile-specific weights $\hat{\omega }(\tau )={\left\{{\hat{\omega }}_{j}(\tau )\right\}}_{j=1}^{p}$ systematically characterize how each variant influences gene expression across the full distribution, capturing regulatory effects that may be concentrated in distributional tails or vary continuously across quantiles.

#### Quantile Weight Integration.

To capture varying genetic effects across quantiles, we develop two complementary strategies for integrating quantile-specific weights $\hat{\omega }(\tau )$ from τ∈{0.01,0.02,…,0.99} based on two quantile region categories, fix regions and dynamic regions. (1) For fix quantile regions, we partition the quantile range into three fixed intervals representing lower (τ∈[0.01,0.33]), middle (τ∈[0.34,0.66]), and upper (τ∈[0.67,0.99]) portions of the expression distribution. (2) For dynamic quantile regions, we use a data-driven approach that automatically determines the number and boundaries of quantile regions based on the correlation structure of $\hat{\omega }(\tau )$ across quantiles. Specifically, we apply hierarchical clustering with modularity optimization^8^ to identify regions $\left\{{𝒜}_{1},\cdots ,{𝒜}_{K}\right\}$ of quantiles with similar generic effect patterns, while ensuring quantile continuity within each cluster (see details in **Supplementary Note S.3**). For each region k, we compute the integrated weight as ${\overset{ˆ}{\omega }}_{k}\;:=\sum_{\tau \in {𝒜}_{k}}\;\hat{\omega }(\tau )$.

#### qTWAS association testing.

For each quantile region k as defined above, the test statistics of Qtwas^9^ is given by

(5) $$Z_{k}=\frac{{\overset{ˆ}{\omega }}_{k}^{⊤}z_{GWAS}}{\sqrt{{\overset{ˆ}{\omega }}_{k}^{⊤}\Sigma {\overset{ˆ}{\omega }}_{k}}},$$

where $z_{\text{GWAS}}$ represents the vector of GWAS z-scores of a disease of interest and Σ denotes the LD matrix across p genetic variants obtained from a custom LD reference panel from the Alzheimer’s Disease Sequencing Project (ADSP), provided by the Genome Center for Alzheimer’s Disease (GCAD)^10^, consisting of approximately 17,000 whole-genome sequenced (WGS) individuals of European ancestry^11^ (see Data availability). Under the null hypothesis of no association between all variants in a gene and disease ($H_{0}\;:\beta_{k}=0),\;Z_{k}$ follows an asymptotic standard normal distribution $\left(Z_{k}\overset{H_{0}\;}{\to }𝒩(0,\;1)\right)$. This approach yields region-specific association statistics that capture distinct patterns of genetic regulation across the gene expression distribution. To obtain a single gene-level association statistic, we combine p-values across all K quantile regions using the Cauchy combination method^6^. Genes with combined p-values below the Bonferroni-corrected threshold across all genes and all contexts are declared significant at a level of 5%^12^.

### Characterize qQTL relative to conventional linear QTL

To characterize the discovery landscape of quantile-specific associations, we directly compare qQTL with linear QTL (xQTL) identified through traditional mean-based methods. Both xQTL and qQTL are identified through marginal *cis*-association testing using identical *cis*-windows and variant sets. For xQTL identification, we perform standard linear regression using Equation (S.10). For qQTL identification, we employ the quantile rank score test under model introduced in Equation (1), evaluating associations across 19 equidistant quantile levels (τ∈{0.05,0.10,…,0.95}). Statistical signficance for both xQTL and qQTL are determined using a unified hierarchical multiple-testing correction procedure (see details in **Supplementary Note S.1**).

At the variant level, we define three mutually categories to characterize the relationship between qQTL and xQTL discoveries:

- “qQTL-only”: significant in qQTL identification but not in xQTL identification,
- “xQTL-only”: significant in xQTL identification but not in qQTL identification,
- “shared QTLs”: significant in both qQTL and xQTL identifications.

To further characterize “shared QTLs”, we assess quantile-specific effect heterogeneity using the Chatterjee correlation test^13^ on quantile-specific regression coefficients $\{\overset{ˆ}{\beta }(\tau ){\}}_{\tau =0.10}^{0.90}$ after LD clumping ($R^{2}<0.8$). ‘shared QTLs” with heterogeneity test p-value < 0.05 are classified as shared-heterogeneous qQTLs, indicating quantile-dependent genetic effects; the remaining “shared QTLs” are classified as shared-homogeneous qQTLs, reflecting consistent effects across the distribution. We use the term “heteogeneous qQTL” (qQTL-het) to collectively refer to “qQTL-only” and shared-heterogeneous associations, representing regulatory effects characterized by distributional heterogeneity.

For all identified qQTL, we characterize genetic effect patterns across the phenotype distribution. Local effects are identified when variants exhibit statistical significance exclusively in upper (τ>0.7) or lower (τ<0.3) quantile regions. To quantify effect heterogeneity, we compute a heterogeneity index defined as $log|sd(\overset{ˆ}{\beta }(\tau ))/mean(\overset{ˆ}{\beta }(\tau ))|$, where $\overset{ˆ}{\beta }(\tau )$ represents the quantile-specific effects across all evaluated quantiles τ, with larger values indicating greater heterogeneity across the gene expression distribution. At the gene level, qQTL genes and xQTL genes are defined as genes harboring at least one significant qQTL or xQTL association, respectively.

### Characterize qQTL relative to conventional non-linear QTL

We characterized the relationship between qQTLs and two major classes of non-linear genetic effects, variance QTLs (vQTLs) and interaction QTLs (iQTLs), to understand how quantile regression captures distributional heterogeneity arising from variance modulation and context-dependent regulation.

### Variance QTLs as a subset of quantile QTLs

We identify vQTLs using a quantile integral linear model (QUAIL)^14^, which tests for genetic effects on phenotypic variance by considering the quantile regression slopes β(τ) across a grid of quantile levels and summarizes variance effects through integrating the contrast $\beta_{j}(\tau )-\beta_{j}(1-\tau )$. In this study, we used 19 quantile levels (τ={0.05,0.10,…,0.95}) for QUAIL, and vQTL significance was determined using hierarchical multiple testing correction^4^ (see details in **Supplementary Note S.1.**)

A genetic effect on variance typically shift the lower and upper tail of phenotypic distribution. In particular, a variant that increases variance tends to decrease the effect estimates at lower quantiles (e.g., τ=0.10or0.25) and increase the effect estimates at upper quantiles (e.g., τ=0.75or0.90), even when the mean expression remains unchanged. QUAIL explicitly targets this pattern by aggregating the differences between lower- and upper-tail quantile co-efficients, and achieves higher power than classical variance-heterogeneity tests. In contrast, our qQTL framework tests each quantile level separately and combines the p-values across multiple quantiles. From this perspective, any vQTL should also correspond to a qQTL with at least one non-zero quantile effect, particularly at the lower and upper tails where variance differences are most pronounced. This theoretical relationship naturally explains our empirical observation that the majority of significant vQTLs were also detected as qQTLs.

### Overlap between interaction QTL and quantile QTL

#### iQTL Mapping.

Interaction QTL (iQTL) capture gene–gene (G×G) or gene–environment (G×E) effects through an explicit interaction between a genetic variant and an interacting factor. We mapped iQTLs by fitting a linear regression model

(6) $$Y_{i}=\beta_{0}+\beta_{G}G_{ij}+\beta_{F}F_{i}+\beta_{I}\left(G_{ij}\times F_{i}\right)+C_{i}^{⊤}\beta_{C}+\epsilon_{i},$$

where $Y_{i}$ is the gene expression, $G_{ij}$ is a genetic variant, $F_{i}$ is an interacting factor, which can be either genetic or environmental, and $C_{i}$ represents additional covariates. Variant–gene pairs were classified as iQTLs if the interaction effect $\beta_{I}$ was statistically significant.

In this study, we performed iQTL mapping for two classes of environmental variables: sex and *APOE ε*4 genotype (discrete) across all datasets and cell-type proportions (continuous) of six major brain cell types (details in Supplementary Note S.1). To avoid potential outlier effects, we applied quantile normalisation to the response variable and restricted the analysis to variants with MAF>0.05. (Other details in Molecular QTL and GWAS data sources). iQTL significance was determined using hierarchical multiple testing correction^4^ (see details in Supplementary Note S.1), controlling hierarchical multiple test FDR at < 0.05 for continuous variables and < 0.25 for discrete variables.

### iQTL directionality and qQTL overlap.

iQTLs can manifest as qQTL signals in marginal quantile regression, even though the interacting factor F or the interaction term G×F. This overlap arises when interaction effects modify the distribution of gene expression across genotypes in a way that shifts particular quantiles, with the pattern determined by the direction of the interaction and the population distribution of F. A detailed description of how gene-gene (GxG) or gene-environment (GxE) interaction effects give rise to marginal qQTL signals are provided in Supplementary Note X. To better understand how interaction effects contribute to marginal qQTL signals, we classified the direction of iQTL effects for continuous environmental variables (cell-type proportions) into three categories based on the effect-size patterns of the linear interaction model:

- “Amplifying”: QTL effect size increases with higher environmental exposure;
- “Counteracting”: QTL effect size decreases with higher environmental exposure;
- “Uncertain”: the genotype main effect is not statistically significant such that its direction cannot be reliably determined.

Specifically, iQTLs with a nominally non-significant genotype main effect ($p_{G}>0.05$) were assigned to the “Uncertain” group, whereas those with a nominally significant main effect ($p_{G}>0.05$) were assigned to the “Amplifying” or “Counteracting” group depending on whether the main effect and interaction effect had the same or opposite signs. This classification draws on prior evidence that “uncertain” iQTLs exhibit poor reproducibility and are likely false-positive results, while “Amplifying” and “Counteracting” iQTLs may arise from distinct biological mechanisms^15^. We therefore examine iQTL–qQTL overlap separately within each directional class.

To understand the relationship between iQTL and qQTL detection, we conducted simulation studies to assess how iQTLs with different directions overlap with qQTL discoveries. To mimic real-world data, we calibrated the simulation settings and effect-size parameters using estimates obtained from fitting APOE *ε*4 QTL. For individual i=1,…,n with n=400, we generate generate $Y_{i}$ from model (6), where the genotype $G_{i}$ and the interaction factor $F_{i}$ coded as {0,1,2} according to Hardy-Weinberg Equilibrium probabilities $\left\{(1-p{)}^{2},2p(1-p),p^{2}\right\}$, using MAF $p_{G}=0.1$ and $p_{F}=0.15$. We include a 45 × 1 covariate vector $C_{i}∼𝒩(0,I)$ and residual $\epsilon_{i}∼𝒩(0,\;0.2667)$. We set the main effect $\beta_{G}=\{0,\;0.15,\;0.3\}$, $\beta_{F}=0$, and interaction effect $\beta_{I}=\{0,\pm 0.1,\ldots ,\pm 0.8\}$, and $\beta_{C}$ fixed to the estimation of regress Y to C in the APOE *ε*4 QTL example. For each simulation configuration, we ran 1,000 Monte Carlo replicates and reported the proportion of variants detected as iQTL, qQTL, or xQTL at significance thresholds $\alpha =\left\{0.05,10^{-6}\right\}$. In addition, focusing on amplifying iQTLs, we summarized across configurations the average proportion of qQTLs that showed evidence of quantile heterogeneity, defined as a significant Chatterjee correlation test^13^ at the same threshold used for QTL detection (i.e. $p_{\xi }<0.05$ for α=0.05 and $p_{\xi }<10^{-6}$ for $\alpha =10^{-6}$)

### Molecular QTL and GWAS data sources

#### Molecular QTL from FunGen-xQTL atlas

We analyze molecular quantitative trait loci (xQTL) generated by the ADSP Functional Genomics Consortium (FunGen–AD), which aggregates deeply phenotyped aging and Alzheimer’s disease cohorts across multiple brain regions, cellular contexts, and molecular modalities. The FunGen–xQTL atlas provides harmonized genotype data and multi-omic phenotypes processed through a unified quality-control and standardization workflow, ensuring cross-cohort comparability. In this study, molecular phenotypes from different cohorts are derived from standardized pipelines for alignment, quantification, and normalization, and all genotype data underwent stringent sample- and variant-level QC, ancestry inference, relatedness filtering, and imputation, followed by harmonization to shared genomic references (GRCh38, dbSNP 151, ENSEMBL v103) through the FunGen–AD data standardization group. To evaluate qQTL identification, we use the molecular contexts across different cohorts shown in Table S1. All xQTLs are computed using the same covariate structure, including fixed covariates, genotype principal components, and phenotype-derived hidden factors with the number of PCs determined by the Marchenko-Pastur limit^16^. And we compute xQTL using an identical univariate association model implemented in TensorQTL^17^, yielding directly comparable, harmonized eQTL and pQTL summary statistics across tissues and cell types.

### GWAS summary statistics for Alzheimer’s disease and aging

We utilize publicly available European-ancestry GWAS summary statistics for Alzheimer’s disease (AD) from three large case-control meta-analyses representing complementary study designs and sample compositions: (1) Primary AD meta-analysis (111,326 cases and 677,663 controls) and its European Alzheimer & Dementia Biobank (EADB) subset (20,301 cases and 21,839 controls) from Bellenguez et al. 2022^18^. (2) AD meta-analysis, which encompasses both clinical and proxy AD cases (90,338 cases in total, including 46,613 proxy cases, and 1,036,225 controls) from Wightman et al. 2021^19^. (3) Clinical/autopsy-confirmed AD meta-analysis (35,274 cases and 59,163 controls) from Kunkle et al. 2019^20^. These datasets represent the major large-scale AD genetic resources currently used for mechanistic and post-GWAS functional analyses and together provide a comprehensive view of AD genetic susceptibility across multiple study designs. For aging-related traits, we use telomere-length GWAS summary statistics based on the first principal component of combined qPCR- and TelSeq-derived measurements from the UK Biobank (TL1; N=462,666 European participants). This dataset provides a high-sample-size genetic summary measure of variation in leukocyte telomere length, serving as a molecular marker of cellular aging processes. For qTWAS identification, we construct a custom LD reference panel from the Alzheimer’s Disease Sequencing Project (ADSP), consisting of approximately 17,000 whole-genome sequenced (WGS) individuals of European ancestry^11^.

### Functional validation and disease heritability enrichment

#### EOO enrichment analysis of functional annotations

To quantify functional differences between two sets of variant–gene links, we use an enrichment-based excess-of-overlap (EOO) analysis^21^. For two link sets $VG_{1}$ and $VG_{2}$, let $\left|VG_{1}\right|$ and $\left|VG_{2}\right|$ denote the number of significant variant–gene (VG) pairs identified by each method, out of $T_{1}$ and $T_{2}$ total tested VG pairs, respectively. Let $T_{12}$ be the number of VG pairs tested by both methods in the same genomic context. We define the EOO statistic as

(7) $$EOO\left(VG_{1},VG_{2}\right)=\frac{\left|VG_{1}\;\cap \;VG_{2}\right|/T_{12}}{\left(\left|VG_{1}\right|/T_{1}\right)\left(\left|VG_{2}\right|/T_{2}\right)},$$

so that EOO > 1 indicates more overlap than expected under independence. Standard errors of EOO are obtained using a leave-one-chromosome-out strategy, and enrichment values are summarized per context.

We apply EOO to evaluate enrichment of VG links in a comprehensive panel of genomic elements. Specifically, we consider approximately 1,500 annotations grouped into 21 functional categories, including 1,226 elements curated from FILER^22^, 97 baselineLD (v2.2) functional annotations^23,24^, STARR-seq annotations^25^, and 105 ENCODE^26^-derived resources. For each molecular context, we construct two VG link sets from marginal *cis*-association results after hierarchical multiple-testing control (hierarchical FDR < 0.05): a qQTL-only set (significant by qQTL but not by conventional xQTL) and an xQTL set (significant by xQTL). To test whether enrichments differed between the qQTL-only and xQTL VG sets within the same context, we perform a two-sided *z*-test on the difference of enrichment estimates and controlled for multiple comparisons across annotations using Benjamini–Hochberg FDR^27^ at 0.05. For each context, we report annotations with significant enrichment differences, and highlighted the top five annotations showing higher enrichment in xQTL relative to qQTL-only links. More detailed description of metrics can be found in **Supplementary Note S.5**.

### S-LDSC heritability analysis of qQTL and xQTL annotations

To evaluate how qQTL and xQTL signals contribute to complex trait heritability, we construct binary SNP annotations from marginal cis-association results after hierarchical FDR control (<0.05). For each molecular context, we define two annotations: (1) a *qQTL* annotation, indicating SNPs linked to significant qQTLs; and (2) an *xQTL* annotation, indicating SNPs linked to significant xQTLs. Each annotation records whether a SNP is linked to at least one significant variant–gene pair in the corresponding set.

We apply stratified LD score regression (S-LDSC) jointly with the 97-category baseline-LD v2.2 model, using approximately 8 million autosomal SNPs and LD scores computed from the ADSP reference panel. For each annotation a, S-LDSC estimates the per-SNP heritability contribution $\tau_{a}$ from the regression $E[\chi_{j}^{2}]=1+\sum_{a}\;\tau_{a}\ell_{j,a}+\epsilon_{j}$, where $\ell_{j,a}$ is the LD score of SNP j with respect to annotation a. We report two quantities for each annotation and context: (1) *marginal enrichment*, defined as

(8) $${\text{Enrichment}}_{a}=\frac{h_{g,a}^{2}/h_{g}^{2}}{M_{a}/M},$$

where $h_{g,a}^{2}$ is the heritability explained by SNPs in annotation a, $M_{a}$ is the number of SNPs in the annotation, and M is the total number of SNPs; and (2) the standardized effect size for annotation a, defined by normalizing the per-SNP heritability coefficient $\tau_{a}$ by the mean per-SNP heritability as

(9) $$\tau_{a}^{*}=\frac{\tau_{a}\;sd(a)}{\sum_{j}\;\;Var\left(\beta_{j}\right)/M},$$

where sd(a) is the standard deviation of the annotation values across all SNPs, $\beta_{j}$ is the per-variant effect size, and $h_{g}^{2}=\sum_{j}\;Var\left(\beta_{j}\right)$ denotes the total SNP-heritability across all annotations so that $\sum_{j}\;Var\left(\beta_{j}\right)/M$ represents the mean per-SNP heritability. Here, $\tau_{a}^{*}$ is interpreted as the proportionate change in per-SNP heritability associated with a one-standard-deviation increase in annotation a, conditional on all other annotations included in the model. To quantify the independent contributions of qQTL-only and xQTL signals, we additionally perform joint-annotation S-LDSC by including both annotations simultaneously—with the 97 baseline annotations—in the same model. Standard errors and *p*-values are obtained via the LDSC block jackknife (200 blocks). Full methodological details are provided in **Supplementary Note S.6.**

### KEGG and Gene Ontology enrichment analysis

To assess whether predefined sets of genes are preferentially involved in known biological pathways or functional categories, we perform over-representation analyses using the KEGG pathway database^28^ and the Gene Ontology (GO)^29^. The goal of this enrichment analysis is to test whether a given gene set contains more members of a pathway or GO term than expected under a genome-wide background. Let 𝒰 denote the universe of background genes considered in the analysis. Denote 𝒢⊂𝒰 as a gene set of interest and 𝒯⊂𝒰 from a given pathway or GO term. Consider a 2 × 2 contingency table, enrichment is defined as

(9) $$\text{Enrichment}(𝒢,𝒯)=\frac{\left|𝒢\cap 𝒯|\;\times \;|𝒰\backslash (𝒢\cup 𝒯)\right|}{\left|𝒢\backslash 𝒯|\;\times \;|𝒯\backslash 𝒢\right|},$$

where, for example, |𝒢\𝒯| represents genes in 𝒢 but not in 𝒯. Enrichment *p*-values are computed using the standard hypergeometric over-representation test. Multiple testing across all pathways and GO terms is controlled using the Benjamini–Hochberg FDR, and terms with FDR-adjusted *p*-values below a specified threshold are considered significantly enriched.

For KEGG, we use the human pathway collection (organism = “hsa”) to evaluate enrichment of signaling, metabolic, and disease-related pathways. For Gene Ontology, enrichment is performed separately for the three GO domains—Biological Process (BP), Cellular Component (CC), and Molecular Function (MF)—to capture complementary aspects of gene function, cellular localization, and molecular activity. Gene identifiers are harmonized to Entrez Gene IDs prior to analysis, and genes without valid mappings are excluded from the testing universe.

## Supplementary Material

SUPPLEMENTARY NOTE & FIGURES

https://www.overleaf.com/project/652803dda9318d04d5f46393

SUPPLEMENTARY TABLES

1. Supplementary Table 1 - Cohort Statistics

2. Supplementary Table 2 - List of model features

3. Supplementary Table 3 - eMAGMA results - predicted eQTLs

4. Supplementary Table 4 - eMAGMA results - finemapped eQTLs

5. Supplementary Table 5 - MAGMA results

6. Supplementary Table 6 - Summary Statistics for BLNK Locus

## Figures and Tables

**Figure 6:**
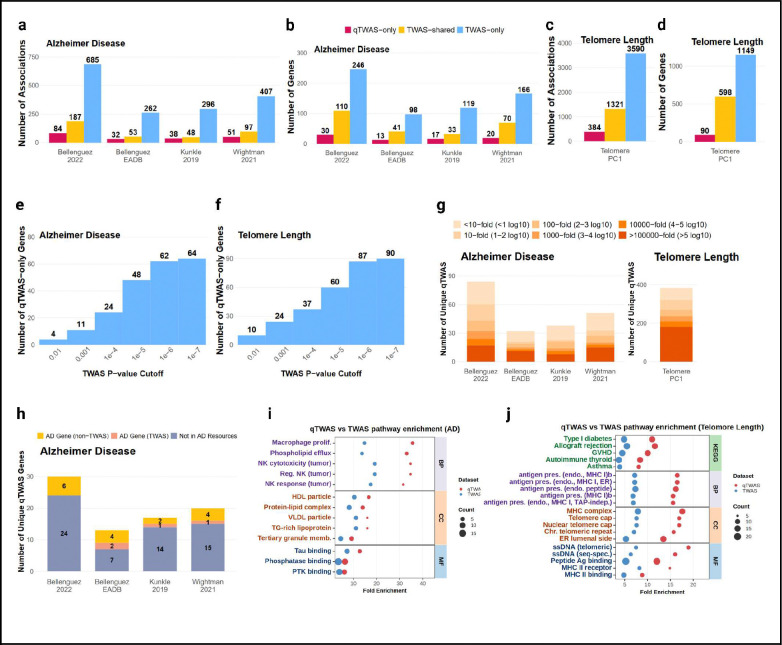
qTWAS discovery on disease-relevant gene and context-specific associations for Alzheimer’s disease and telomere length. Analyses were performed across four Alzheimer’s disease (AD) GWAS studies (Bellenguez 2022, EADB 2022, Kunkle 2019, Wightman 2021) and one telomere length GWAS (telomere length PC1). Significance was defined as Bonferroni-adjusted p < 0.05 across molecular contexts. **a–b**, Counts of significant gene–context associations **(a)** and significant genes **(b)** stratified by qTWAS-only (red), TWAS-shared (gold), and TWAS-only (blue) in the four AD GWAS studies. **c–d**, Telomere length analyses (analogous to AD panels): counts of significant gene–context associations **(c)** and significant genes **(d). e–f,** Distribution of qTWAS-only genes across TWAS p-value cutoffs for AD **(e)** and telomere length **(f)**. **g**, Fold-change in statistical significance for qTWAS-only associations relative to TWAS, shown as binned increases in −log_10_(*P*) (light to dark orange indicates larger fold-change bins). **h,** Functional genomics evidence for AD qTWAS-only genes, compared against multi-context colocalization, multi-trait fine-mapping, and the complete TWAS catalog from the FunGen-xQTL resource (a harmonized multi-context xQTL database). **i–j**, Pathway enrichment comparison using all significant qTWAS genes versus all significant TWAS genes. Bubble plots show the top five pathways where qTWAS has higher fold enrichment than TWAS for AD **(i)** and telomere length **(j)**. GO categories include Biological Process (BP), Cellular Component (CC), and Molecular Function (MF); KEGG denotes curated pathway sets.
